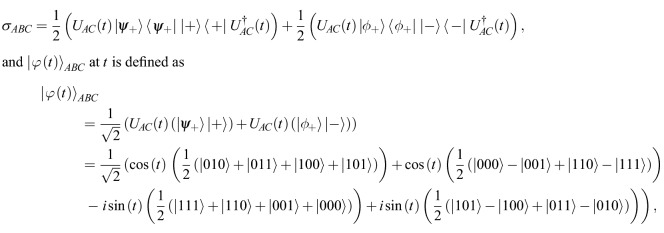# Author Correction: Entanglement Availability Differentiation Service for the Quantum Internet

**DOI:** 10.1038/s41598-020-75950-5

**Published:** 2021-01-06

**Authors:** Laszlo Gyongyosi, Sandor Imre

**Affiliations:** 1 School of Electronics and Computer Science, University of Southampton, Southampton, SO17 1BJ UK; 2 Department of Networked Systems and Services, Budapest University of Technology and Economics, Budapest, 1117 Hungary; 3 MTA-BME Information Systems Research Group, Hungarian Academy of Sciences, Budapest, 1051 Hungary

Correction to: *Scientific Reports* 10.1038/s41598-018-28801-3, published online 13 July 2018

This Article contains errors.

In Protocol 0, where 
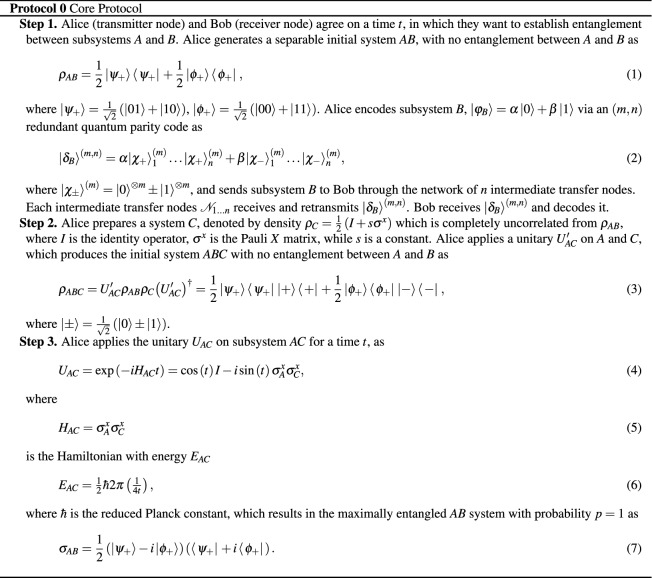


should read: 
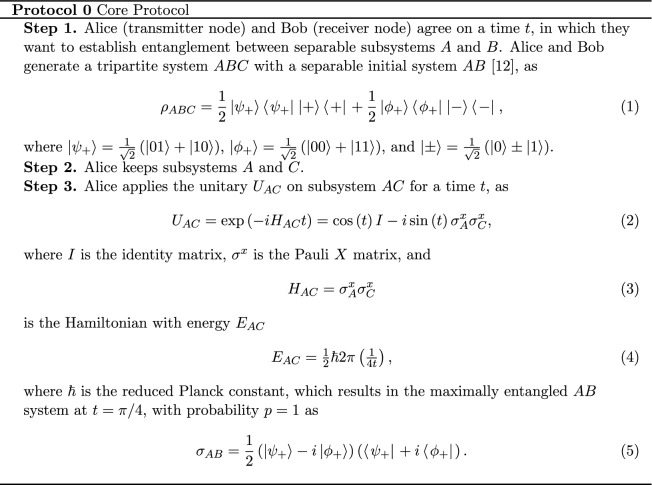


In addition, in Equation 13 and 14

$$\begin{aligned} {\sigma }_{ABC}= & {} |{\varphi }(t)\rangle {\langle {\varphi }(t)|}_{ABC}=U{\rho }_{0}{U}^{\dagger }\\= & {} \frac{{{1}}}{{{2}}}({U}_{AC}|{\psi }_{+}\rangle \langle {\psi }_{+}||+\,\rangle \langle +|{U}_{AC}^{\dagger })+\frac{{{1}}}{{{2}}}({U}_{AC}|{{\phi }}_{+}\rangle \langle {{\phi }}_{+}||\,-\,\rangle \langle -|{U}_{AC}^{\dagger }), \end{aligned}$$

where ${|{\varphi }(t)\rangle }_{ABC}$ at time *t* is evaluated as

$$\begin{aligned} {|{\varphi }(t)\rangle }_{ABC}= &\, {} \frac{{{1}}}{\sqrt{{{2}}}}(\cos (t)(|{\psi }_{+}\rangle |\,+\,\rangle )-i\,\sin (t)(|{{\phi }}_{+}\rangle |\,+\,\rangle ))\\&+\,\frac{{{1}}}{\sqrt{{{2}}}}(\cos (t)(|{{\phi }}_{+}\rangle |\,-\,\rangle )+i\,\sin (t)(|{\psi }_{+}\rangle |\,-\,\rangle ))\\= &\, {} \frac{{{1}}}{\sqrt{{{2}}}}(\cos (t)(|{\psi }_{+}\rangle )-i\,\sin (t)(|{{\phi }}_{+}\rangle ))|\,+\,\rangle \\&+\,\frac{{{1}}}{\sqrt{{{2}}}}(\cos (t)(|{{\phi }}_{+}\rangle )+i\,\sin (t)(|{\psi }_{+}\rangle ))|\,-\,\rangle \end{aligned}$$

should read:

$$\sigma _{{ABC}} = \frac{{1}}{2}\left( {U_{{AC}} (t)|\psi _{ + } \rangle \langle \psi _{ + } || + \rangle \langle + |U_{{AC}}^{{{{\dag}}}} (t)} \right) + \frac{1}{2}\left( {U_{{AC}} (t)|\phi _{ + } \rangle \langle \phi _{ + } || - \rangle \langle - |U_{{AC}}^{{{{\dag}}}} (t)} \right),$$

while ${|{\varphi }(t)\rangle }_{ABC}$ at time *t* is defined as

$$\begin{aligned} | & \varphi (t)\rangle _{{ABC}} \\ & = \frac{{\text{1}}}{{\sqrt {\text{2}} }}(U_{{AC}} (t)(|\psi _{ + } \rangle | + \rangle ) + U_{{AC}} (t)(|\phi _{ + } \rangle | - \rangle )) \\ & = \frac{{\text{1}}}{{\sqrt {\text{2}} }}(\cos (t)(|\psi _{ + } \rangle | + \rangle ) - i\sin (t)(|\phi _{ + } \rangle | + \rangle )) + \frac{{\text{1}}}{{\sqrt {\text{2}} }}(\cos (t)(|\phi _{ + } \rangle | - \rangle) + i\sin (t)(|\psi _{ + } \rangle | - \rangle )) \\ & = \frac{{\text{1}}}{{\sqrt {\text{2}} }}(\cos (t)(|\psi _{ + } \rangle ) - i\sin (t)(|\phi _{ + } \rangle ))| + \rangle + \frac{{\text{1}}}{{\sqrt {\text{2}} }}(\cos (t)(|\phi _{ + } \rangle ) + i\sin (t)(|\psi _{ + } \rangle ))| - \rangle \\ \end{aligned}$$

Furthermore, in Equation 22 and 23

$$\begin{aligned} {\sigma }_{ABC}= & \,{} |{\varphi }(t)\rangle {\langle {\varphi }(t)|}_{ABC}=U{\rho }_{0}{U}^{\dagger }\\= & \,{} \frac{{{1}}}{{{2}}}({U}_{AC}|{\psi }_{+}\rangle \langle {\psi }_{+}||\,+\,\rangle \langle +|{U}_{AC}^{\dagger })+\frac{{{1}}}{{{2}}}({U}_{AC}|{{\phi }}_{+}\rangle \langle {{\phi }}_{+}||\,-\,\rangle \langle -|{U}_{AC}^{\dagger }), \end{aligned}$$

where ${|\varphi (t)\rangle }_{ABC}$ at *t* is evaluated as

$$\begin{aligned} {|{\varphi }(t)\rangle }_{ABC}= & \,{} \frac{{{1}}}{\sqrt{{{2}}}}({U}_{AC}(|{\psi }_{+}\rangle |+\rangle )+{U}_{AC}(|{{\phi }}_{+}\rangle |-\rangle ))\\= & \,{} \frac{{{1}}}{\sqrt{{{2}}}}\left( \cos (t)\left( \frac{{{1}}}{{{2}}}(|{{010}}\rangle +|{{011}}\rangle +|{{100}}\rangle +|{{101}}\rangle )\right) +{{cos}}(t)\left( \frac{{{1}}}{{{2}}}(|000\rangle -|{{001}}\rangle +|{{110}}\rangle -|{{111}}\rangle )\right) \right. \\&-\,i\,\sin (t)\left( \frac{{{1}}}{{{2}}}(|{{111}}\rangle +|{{110}}\rangle +|{{001}}\rangle +|000\rangle )\right) \\&\left. +\,i\,\sin (t)\left( \frac{{{1}}}{{{2}}}\left( |{{101}}\rangle -|{{100}}\rangle +|{{011}}\rangle -|{{010}}\rangle \right) \right) \right) \\= & \,{} \frac{{{1}}}{\sqrt{{{2}}}}(\cos (t)(|{\psi }_{+}\rangle |+\rangle +|{{\phi }}_{+}\rangle |\,-\rangle )-i\,\sin (t)(|{{\phi }}_{+}\rangle |+\rangle -|{\psi }_{+}\rangle |-\rangle ))\\= & \,{} \frac{{{1}}}{\sqrt{{{2}}}}((\cos (t)(|{\psi }_{+}\rangle )-i\,\sin (t)(|{{\phi }}_{+}\rangle ))|\,+\,\rangle +(\cos (t)(|{{\phi }}_{+}\rangle )+i\,\sin (t)(|{\psi }_{+}\rangle ))|\,-\,\rangle ), \end{aligned}$$

should read:

$$\sigma _{{ABC}} = \frac{{\text{1}}}{{\text{2}}}\left( {U_{{AC}} (t)|\psi _{ + } \rangle \langle \psi _{ + } || + \rangle \langle + |U_{{AC}}^{\dag } (t)} \right) + \frac{{\text{1}}}{{\text{2}}}\left( {U_{{AC}} (t)|\phi _{ + } \rangle \langle \phi _{ + } || - \rangle \langle - |U_{{AC}}^{\dag } (t)} \right),$$

and ${|\varphi (t)\rangle }_{ABC}$ at *t* is defined as

$$\begin{aligned} & |\varphi (t)\rangle _{{ABC}} \\ & = \frac{{\text{1}}}{{\sqrt {\text{2}} }}(U_{{AC}} (t)(|\psi _{ + } \rangle | + \rangle ) + U_{{AC}} (t)(|\phi _{ + } \rangle | - \rangle )) \\ & = \frac{{\text{1}}}{{\sqrt {\text{2}} }}\left( {\cos (t)\left( {\frac{{\text{1}}}{{\text{2}}}(|{\text{010}}\rangle + |{\text{011}}\rangle + |{\text{100}}\rangle + |{\text{101}}\rangle )} \right)} \right. + {\text{cos}}(t)\left( {\frac{{\text{1}}}{{\text{2}}}(|000\rangle - |{\text{001}}\rangle + |{\text{110}}\rangle - |{\text{111}}\rangle )} \right) \\ & - i \sin (t)\left( {\frac{{\text{1}}}{{\text{2}}}(|{\text{111}}\rangle + |{\text{110}}\rangle + |{\text{001}}\rangle + |000\rangle )} \right)\left. { + i \sin (t)\left( {\frac{{\text{1}}}{{\text{2}}}(|{\text{101}}\rangle - |{\text{100}}\rangle + |{\text{011}}\rangle - |{\text{010}}\rangle )} \right)} \right) \\ & = \frac{{\text{1}}}{{\sqrt {\text{2}} }}(\cos (t)(|\psi _{ + } \rangle | + \rangle + |\phi _{ + } \rangle | - \rangle ) - i \sin (t)(|\phi _{ + } \rangle | + \rangle - |\psi _{ + } \rangle | - \rangle )) \\ & = \frac{{\text{1}}}{{\sqrt {\text{2}} }}((\cos (t)(|\psi _{ + } \rangle ) - i \sin (t)(|\phi _{ + } \rangle ))| + \rangle + (\cos (t)(|\phi _{ + } \rangle ) + i \sin (t)(|\psi _{ + } \rangle ))| - \rangle ), \\ \end{aligned}$$

Finally, in equation A.3 and A.4 in the Supplementary Information file, 
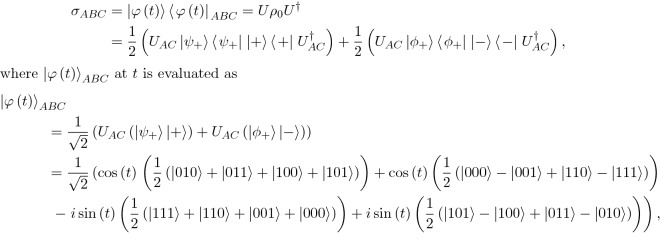


should read: