# Supplementary material for: Entanglement Availability Differentiation Service for the Quantum Internet
Source: Sci Rep. 2018 Jul 13;8:10620. doi: 10.1038/s41598-018-28801-3 (PMC6045587; doi:10.1038/s41598-018-28801-3)
Supplement: Supplementary file 1 — Supplementary Information [file 41598_2018_28801_MOESM1_ESM.pdf]

Supplemental Information

Entanglement Availability Differentiation Service for the Quantum Internet

L. Gyongyosi*[a,b,c] and S. Imre[b]

[a] School of Electronics and Computer Science
University of Southampton, Southampton SO17 1BJ, UK;
[b] Department of Networked Systems and Services, Budapest University of Technology and Economics, Budapest, Hungary;
[c] MTA-BME Information Systems Research Group, Hungarian Academy of Sciences, H-1518, Budapest, Hungary
[*] l.gyongyosi@soton.ac.uk

Abstract

A fundamental concept of the quantum Internet is quantum entanglement. In a quantum Internet scenario where the legal users of the network have different priority levels or where a differentiation of entanglement availability between the users is a necessity, an entanglement availability service is essential. Here we define the entanglement availability differentiation (EAD) service for the quantum Internet. In the proposed EAD framework, the differentiation is either made in the amount of entanglement with respect to the relative entropy of entanglement associated with the legal users, or in the time domain with respect to the amount of time that is required to establish a maximally entangled system between the legal parties. The framework provides an efficient and easily-implementable solution for the differentiation of entanglement availability in experimental quantum networking scenarios.

Acknowledgements

L.GY. would like to thank Tomasz Paterek for useful discussions. This work was partially supported by the National Research Development and Innovation Office of Hungary (Project No. 2017-1.2.1-NKP-2017-00001), by the Hungarian Scientific Research Fund - OTKA K-112125 and in part by the BME Artificial Intelligence FIKP grant of EMMI (BME FIKP-MI/SC).

A Appendix

A.1 Steps of the Core Protocol

The detailed discussion of the Core Protocol (Protocol 0) is as follows.

In Step 1, the input system AB (1) is an even mixture of the Bell states which contains no entanglement. It is also the situation in Step 2 for the subsystem AB of ρ_{ABC} (3), thus the relative entropy of entanglement for ρ_{AB} is zero, $E(A:B) = 0$. The initial ρ_{AB} in (1) and (3), is the unentangled, Bell-diagonal state

$$\rho_{AB} = \tfrac{1}{4}\begin{pmatrix} 1 & 0 & 0 & 1 \\ 0 & 1 & 1 & 0 \\ 0 & 1 & 1 & 0 \\ 1 & 0 & 0 & 1 \end{pmatrix} \tag{A.1}$$

with eigenvalues $v_+ = \frac{1}{2}, v_- = 0, u_+ = \frac{1}{2}, u_- = 0$.

In Step 3, dynamics generated by local Hamiltonian $H_{AC} = \sigma_A^x \sigma_C^x$ with energy E_{AC} will lead to entanglement oscillations in AB. Thus, if U_{AC} is applied exactly only for a well determined time t, the local unitary will lead to maximally entangled AB with a unit probability.

As a result, for subsystem AB, the entanglement $E(A:B)$ oscillates [10] with the application time t of the unitary. In particular, the entanglement oscillation in AB generated by the energy E_{AC} (6) of the Hamiltonian H_{AC} (5). This oscillation has a period time T_π, which exactly equals to $4t$, thus

$$T_\pi = 4t, \tag{A.2}$$

where t is determined by Alice and Bob. In other words, time t identifies $\pi/4$, where π is the oscillation period.

Therefore, after Step 3, the density σ_{ABC} of the final ABC state is as

$$\begin{aligned} \sigma_{ABC} &= |\varphi(t)\rangle \langle \varphi(t)|_{ABC} = U\rho_0 U^\dagger \\ &= \frac{1}{2}\left(U_{AC}|\psi_+\rangle\langle\psi_+|\,|+\rangle\langle+|\,U_{AC}^\dagger\right) + \frac{1}{2}\left(U_{AC}|\phi_+\rangle\langle\phi_+|\,|-\rangle\langle-|\,U_{AC}^\dagger\right), \end{aligned} \tag{A.3}$$

where $|\varphi(t)\rangle_{ABC}$ at t is evaluated as

$$\begin{aligned} &|\varphi(t)\rangle_{ABC} \\ &\quad= \frac{1}{\sqrt{2}}\left(U_{AC}(|\psi_+\rangle|+\rangle) + U_{AC}(|\phi_+\rangle|-\rangle)\right) \\ &\quad= \frac{1}{\sqrt{2}}\left(\cos(t)\left(\frac{1}{2}(|010\rangle + |011\rangle + |100\rangle + |101\rangle)\right) + \cos(t)\left(\frac{1}{2}(|000\rangle - |001\rangle + |110\rangle - |111\rangle)\right)\right. \\ &\quad\left. - i\sin(t)\left(\frac{1}{2}(|111\rangle + |110\rangle + |001\rangle + |000\rangle)\right) + i\sin(t)\left(\frac{1}{2}(|101\rangle - |100\rangle + |011\rangle - |010\rangle)\right)\right), \end{aligned} \tag{A.4}$$

that can be rewritten as

$$\begin{aligned} &\frac{1}{\sqrt{2}}\left(\cos(t)(|\psi_+\rangle|+\rangle + |\phi_+\rangle|-\rangle) - i\sin(t)(|\phi_+\rangle|+\rangle - |\psi_+\rangle|-\rangle)\right) \\ &= \frac{1}{\sqrt{2}}\left((\cos(t)(|\psi_+\rangle) - i\sin(t)(|\phi_+\rangle))|+\rangle + (\cos(t)(|\phi_+\rangle) + i\sin(t)(|\psi_+\rangle))|-\rangle\right), \end{aligned} \tag{A.5}$$

where the sign change on $U_{AC}\left(\left|\phi_{+}\right\rangle\left|-\right\rangle\right)$ is due to the $\left|-\right\rangle$ eigenstate on C.

Thus, at $t=\pi/4$,

$$\begin{aligned}\left|\varphi\left(\pi/4\right)\right\rangle_{ABC} &= \frac{1}{\sqrt{2}}\left(\cos\left(\pi/4\right)\left(\left|\psi_{+}\right\rangle\left|+\right\rangle+\left|\phi_{+}\right\rangle\left|-\right\rangle\right)-i\sin\left(\pi/4\right)\left(\left|\phi_{+}\right\rangle\left|+\right\rangle-\left|\psi_{+}\right\rangle\left|-\right\rangle\right)\right)\\ &= \frac{1}{\sqrt{2}}\left(\frac{1}{\sqrt{2}}\left(\left|\psi_{+}\right\rangle\left|+\right\rangle+\left|\phi_{+}\right\rangle\left|-\right\rangle\right)-i\frac{1}{\sqrt{2}}\left(\left|\phi_{+}\right\rangle\left|+\right\rangle-\left|\psi_{+}\right\rangle\left|-\right\rangle\right)\right)\\ &= \frac{1}{\sqrt{2}}\left(\begin{array}{c}\left(\frac{1}{\sqrt{2}}\left(\left|\psi_{+}\right\rangle\right)-i\frac{1}{\sqrt{2}}\left(\left|\phi_{+}\right\rangle\right)\right)\left|+\right\rangle\\ +\left(\frac{1}{\sqrt{2}}\left(\left|\phi_{+}\right\rangle\right)+i\frac{1}{\sqrt{2}}\left(\left|\psi_{+}\right\rangle\right)\right)\left|-\right\rangle\end{array}\right),\end{aligned} \tag{A.6}$$

where

$$\frac{1}{\sqrt{2}}\left(\left|\phi_{+}\right\rangle+i\left|\psi_{+}\right\rangle\right)=i\left(\frac{1}{\sqrt{2}}\left(\left|\psi_{+}\right\rangle-i\left|\phi_{+}\right\rangle\right)\right); \tag{A.7}$$

i.e., up to the global phase both states are the same.

Therefore the $\left|\varphi\left(\pi/4\right)\right\rangle_{ABC}$ system state of ABC at $t=\pi/4$ is yielded as

$$\left|\varphi\left(\pi/4\right)\right\rangle_{ABC}=\frac{1}{\sqrt{2}}\left(\frac{1}{\sqrt{2}}\left(\left|\psi_{+}\right\rangle-i\left|\phi_{+}\right\rangle\right)\right)\left|+\right\rangle+\frac{1}{\sqrt{2}}\left(\frac{1}{\sqrt{2}}\left(\left|\psi_{+}\right\rangle-i\left|\phi_{+}\right\rangle\right)\right)\left|-\right\rangle, \tag{A.8}$$

while the density matrix σ_{ABC} of the final ABC system in matrix form is as

$$\sigma_{ABC}=\tfrac{1}{8}\begin{pmatrix}1&0&-i&0&-i&0&1&0\\0&1&0&-i&0&-i&0&1\\i&0&1&0&1&0&i&0\\0&i&0&1&0&1&0&i\\i&0&1&0&1&0&i&0\\0&i&0&1&0&1&0&i\\1&0&-i&0&-i&0&1&0\\0&1&0&-i&0&-i&0&1\end{pmatrix}. \tag{A.9}$$

As one can verify, the resulting AB state $\left|\xi\left(\pi/4\right)\right\rangle_{AB}$ at $t=\pi/4$ is pure and maximally entangled,

$$\left|\xi\left(\pi/4\right)\right\rangle_{AB}=\frac{1}{\sqrt{2}}\left(\left|\psi_{+}\right\rangle-i\left|\phi_{+}\right\rangle\right), \tag{A.10}$$

yielding relative entropy of entanglement

$$E\left(A:B\right)=1 \tag{A.11}$$

with unit probability.

The σ_{AB} density matrix of the final AB state is

$$\begin{aligned}\sigma_{AB} &= \left|\xi(\pi/4)\right\rangle\left\langle\xi(\pi/4)\right|_{AB}\\ &= \frac{1}{2}\left(\left|\psi_{+}\right\rangle-i\left|\phi_{+}\right\rangle\right)\left(\left\langle\psi_{+}\right|+i\left\langle\phi_{+}\right|\right)\\ &= \frac{1}{2}\left(\left|\psi_{+}\right\rangle\left\langle\psi_{+}\right|+i\left|\psi_{+}\right\rangle\left\langle\phi_{+}\right|-i\left|\phi_{+}\right\rangle\left\langle\psi_{+}\right|+\left|\phi_{+}\right\rangle\left\langle\phi_{+}\right|\right),\end{aligned} \tag{A.12}$$

which in matrix form is as

$$\sigma_{AB} = \tfrac{1}{4}\begin{pmatrix} 1 & -i & -i & 1 \\ i & 1 & 1 & i \\ i & 1 & 1 & i \\ 1 & -i & -i & 1 \end{pmatrix}. \tag{A.13}$$

The negativity for the $\sigma_{AB}^{T_B}$ partial transpose of σ_{AB} yields

$$\mathrm{N}\left(\sigma_{AB}^{T_B}\right) = \tfrac{\left\|\sigma_{AB}^{T_B}\right\|-1}{2} = \frac{\mathrm{Tr}\left(\sqrt{\left(\sigma_{AB}^{T_B}\right)^{\dagger}\sigma_{AB}^{T_B}}\right)-1}{2} = \tfrac{i}{2}, \tag{A.14}$$

which also immediately proves that AB is maximally entangled. For a comparison, for the density matrix of initial AB, (1), is $\mathrm{N}\left(\rho_{AB}^{T_B}\right) = 0$.

Note that subsystem C requires no further storage in a quantum memory, since the output density σ_{ABC} can be rewritten as

$$\begin{aligned} \sigma_{ABC} &= \frac{1}{2}\left(\left|\xi\left(\pi/4\right)\right\rangle_{AB}\left|+\right\rangle\right)\left(\left\langle\xi\left(\pi/4\right)\right|_{AB}\left\langle+\right|\right) + \frac{1}{2}\left(\left|\xi\left(\pi/4\right)\right\rangle_{AB}\left|-\right\rangle\right)\left(\left\langle\xi\left(\pi/4\right)\right|_{AB}\left\langle-\right|\right) \\ &= \left|\xi\left(\pi/4\right)\right\rangle\left\langle\xi\left(\pi/4\right)\right|_{AB}\left(\left|+\right\rangle\left\langle+\right| + \left|-\right\rangle\left\langle-\right|\right) \\ &= \left(\left|\xi\left(\pi/4\right)\right\rangle\left\langle\xi\left(\pi/4\right)\right|_{AB}\right) I, \end{aligned} \tag{A.15}$$

where I is the identity operator, therefore the protocol does not require long-lived quantum memories.

A.1.1 Classical Correlations

The classical correlation is transmitted subsystem B of (1) in Step 1 is as follows. Since ρ_{AB} is a Bell-diagonal state [49] of two qubits A and B it can be written as

$$\rho_{AB} = \frac{1}{4}\left(I + \sum_{j=1}^{3} c_j \sigma_j^A \otimes \sigma_j^B\right) = \sum_{a,b} \lambda_{ab}\left|\beta_{ab}\right\rangle\left\langle\beta_{ab}\right|, \tag{A.16}$$

where terms σ_j refer to the Pauli operators, while $|\beta_{ab}\rangle$ is a Bell-state

$$\left|\beta_{ab}\right\rangle = \frac{1}{\sqrt{2}}\left(\left|0,b\right\rangle + (-1)^a\left|1,1\oplus b\right\rangle\right), \tag{A.17}$$

while λ_{ab} are the eigenvalues as

$$\lambda_{ab} = \frac{1}{4}\left(1 + (-1)^a c_1 - (-1)^{a+b} c_2 + (-1)^b c_3\right). \tag{A.18}$$

The $\mathcal{I}$ quantum mutual information of Bell diagonal state ρ_{AB} quantifies the total correlations in the joint system ρ_{AB} as

$$\begin{aligned} \mathcal{I} &= S\left(\rho_A\right) + S\left(\rho_B\right) - S\left(\rho_{AB}\right) \\ &= S\left(\rho_B\right) - S\left(B|\,A\right) \\ &= 2 - S\left(\rho_{AB}\right) \\ &= \sum_{a,b} \lambda_{ab}\log_2\left(4\lambda_{ab}\right), \end{aligned} \tag{A.19}$$

where $S(\rho) = -\mathrm{Tr}(\rho \log_2 \rho)$ is the von Neumann entropy of ρ, and $S(B|A) = S(\rho_{AB}) - S(\rho_A)$ is the conditional quantum entropy.

The $\mathcal{C}(\rho_{AB})$ classical correlation function measures the purely classical correlation in the joint state ρ_{AB}. The amount of purely classical correlation $\mathcal{C}(\rho_{AB})$ in ρ_{AB} can be expressed as follows [49]:

$$\begin{aligned}\mathcal{C}(\rho_{AB}) &= S(\rho_B) - \tilde{S}(B|A) \\ &= S(\rho_B) - \min_{E_k} \sum_k p_k S\left(\rho_{B|k}\right) \\ &= 1 - H\left(\frac{1+c}{2}\right) \\ &= \frac{1+c}{2}\log_2(1+c) + \frac{1-c}{2}\log_2(1-c),\end{aligned} \tag{A.20}$$

where

$$\rho_{B|k} = \frac{\langle k|\rho_{AB}|k\rangle}{\langle k|\rho_A|k\rangle} \tag{A.21}$$

is the post-measurement state of ρ_B, the probability of result k is

$$p_k = Dq_k\langle k|\rho_A|k\rangle, \tag{A.22}$$

while d is the dimension of system ρ_A and the q_k make up a normalized probability distribution, $E_k = Dq_k|k\rangle\langle k|$ are rank-one POVM (positive-operator valued measure) elements of the POVM measurement operator E_k [49], while $H(p) = -p\log_2 p - (1-p)\log_2(1-p)$ is the binary entropy function, and

$$c = \max|c_j|. \tag{A.23}$$

For the transmission of B the subsystem ρ_{AB} is expressed as given by (1), thus the classical correlation during the transmission is

$$\mathcal{C}(\rho_{AB}) = 1 - H\left(\frac{1+c}{2}\right) = 1, \tag{A.24}$$

where $c = 1$.

A.2 Abbreviations

EAD Entanglement Availability Differentiation

POVM Positive-Operator Valued Measure

A.3 Notations

The notations of the manuscript are summarized in Table A.1.

Table A.1: Summary of notations.

Notation	*Description*
ρ_{ABC}	Initial system.
σ_{ABC}	Final system.
ρ_{AB}, ρ_C	Initial subsystems.
$\lvert\delta_B\rangle^{(m,n)}$	Subsystem B, $\lvert\varphi_B\rangle = \alpha\lvert 0\rangle + \beta\lvert 1\rangle$, encoded via an (m, n) redundant quantum parity code as $\lvert\delta_B\rangle^{(m,n)} = \alpha\lvert\chi_+\rangle_1^{(m)} \dots \lvert\chi_+\rangle_n^{(m)} + \beta\lvert\chi_-\rangle_1^{(m)} \dots \lvert\chi_-\rangle_n^{(m)}$, where $\lvert\chi_\pm\rangle^{(m)} = \lvert 0\rangle^{\otimes m} \pm \lvert 1\rangle^{\otimes m}$.
T	Period time selected by Alice and Bob.
$\mathcal{N}_{1\dots n}$	Intermediate quantum repeaters between Alice and Bob.
σ^x	Pauli X matrix.
H_{AC}	Hamiltonian, $H_{AC} = \sigma_A^x \sigma_C^x$.
E_{AC}	Energy of Hamiltonian H_{AC}.
U_{AC}	Unitary, applied by Alice on subsystem AC for a time t, $U_{AC} = \exp(-iH_{AC}t)$, where $H_{AC} = \sigma_A^x \sigma_C^x$ is a Hamiltonian, σ^x is the Pauli X matrix.
t	Application time of unitary U_{AC}, determined by Alice and Bob.
I	Identity operator.
$\hbar$	Reduced Planck constant.
$E(\cdot)$	Relative entropy of entanglement.
T_π	Oscillation period, $T_\pi = 4t$, where π is the period.
$\lvert\xi\left(\frac{\pi}{4}\right)\rangle_{AB}$	Output AB subsystem at time t, $\lvert\xi\left(\frac{\pi}{4}\right)\rangle_{AB} = \frac{1}{\sqrt{2}}(\lvert\psi_+\rangle - i\lvert\phi_+\rangle)$, where $\lvert\psi_+\rangle = \frac{1}{\sqrt{2}}(\lvert 01\rangle + \lvert 10\rangle)$, $\lvert\phi_+\rangle = \frac{1}{\sqrt{2}}(\lvert 00\rangle + \lvert 11\rangle)$ are maximally entangled states.
$\sigma_{AB}^{T_B}$	Partial transpose of output AB subsystem σ_{AB}.
$N\left(\sigma_{AB}^{T_B}\right)$	Negativity for the $\sigma_{AB}^{T_B}$ partial transpose of σ_{AB}.
